# Improving the Living, Learning, and Thriving of Young Black Men: A Conceptual Framework for Reflection and Projection

**DOI:** 10.3390/ijerph16081331

**Published:** 2019-04-13

**Authors:** Daphne C. Watkins

**Affiliations:** School of Social Work, University of Michigan, Ann Arbor, MI 48109, USA; daphnew@umich.edu

**Keywords:** African American/Black men, intervention, manhood, masculinity, mental heath, social media

## Abstract

Black men experience disproportionate mental health challenges due to their exposure to severe psychosocial stressors. Yet, the mental health challenges of Black men have largely been left out of national conversations. Strong theoretical frameworks are important when generating dialogue about the mental health of Black men, as it helps to validate the work on a larger scale while also grounding the work for more practical use. This paper presents the conceptual framework for a five-year initiative aimed at improving the living, learning, and thriving of young Black men through a social media intervention that improves their mental health, expands their definitions of manhood, and helps them to engage in social support. The Young, Black Men, Masculinities, and Mental Health (YBMen) project is a social media-based, health promotion program that targets mental health (e.g., depressive symptoms), masculine norms (e.g., definitions of manhood), and social support for young Black men using culturally-sensitive, age-appropriate, and gender-specific popular culture. The YBMen project has been successfully implemented with over 150+ Black men since 2014; findings demonstrate improved mental health outcomes, progressive definitions of manhood, and stronger social relationships. Reflections from the past and projections for the future are discussed.

## 1. Introduction

As they progress to and through their developmental years, young adults experience changes with their physical and sexual maturity, social relationships, identity, independence, and educational demands [1,2,3], which can prompt health problems, trigger vulnerabilities, and constrain coping behaviors and access to resources. In addition to these developmental changes, young Black men experience additional challenges associated with their exposure to psychosocial stressors compared to young men of other races [4,5,6,7,8]. Decades of research have chronicled Black men’s challenges with human service providers [7,9], racial discrimination [5,6,7,8,9,10,11], the criminal justice system [12,13], racial and cultural identity [7,14,15], depression [4,5,7,8,10,12,16,17], violence [9], and masculine gender norms [7,11,17,18,19,20,21]. Yet, the mental health challenges of young Black men have largely been left out of national conversations. Furthermore, due to the stigma associated with mental health conditions, these conversations rarely occur in Black communities. This barrier can make mental health challenges among Black men difficult to address. Moreover, mortality rates and healthcare system mistrust by Black men may mask their health behaviors and make it difficult to monitor their health as they transition to and through adulthood. Many stressors experienced by young Black men are unique to them and recount deeply held beliefs about what it means to be both Black and male in society [7].

Studies suggest the compounded effects of race and gender may place Black men at disproportionately high rates of disadvantage across a myriad of risks, such as depression, anxiety, and substance misuse [22,23,24,25,26]. When taken together, mental health, masculinities (or manhood), and social support among Black men are grossly understudied, yet research suggests that men who are mentally healthy, have progressive definitions of manhood, and engage in social support have better health status, role functioning, psychosocial adjustment, coping behaviors, quality of life, well-being, and self-actualization [27,28,29,30]. Research has also confirmed the value of culturally relevant, age-appropriate, and gender-specific mental health efforts aimed at Black men [7,31,32,33]. Moreover, grounding programs in theory allows for the testing of program components, builds on previous research, and establishes a framework for program evaluation and sustainability.

The purpose of this paper was to present the conceptual framework for a five-year initiative aimed at improving the living, learning, and thriving of Black men through a social media-based program called the Young Black Men, Masculinities, and Mental Health (YBMen) project. The goal of the YBMen project was to improve young Black men’s mental health, expand their definitions of manhood, and improve their engagement in social support. In this paper, I describe the synergistic framework used to design, implement, and evaluate the YBMen project; reflect on the program’s progress to date; and then project next steps for this and other programs aimed at young Black men and men from other marginalized groups.

## 2. Background

### Mental Health, Masculinities, and Social Support for Young Black Men

Previous studies suggest Black men experience and express mental health conditions in ways that are different compared to Black women or White men [34,35]. For instance, Black men may define depression based on their personal accounts [23,25,26], suggesting Black men may rely on terminology not in the DSM-5 to frame their knowledge, attitudes, and beliefs about depression. Likewise, the origins of Black men’s responses to depression and distress are different compared to those of other men and are described in the context of their families, work, school, and communities [7,11,14,21,22]. For example, rather than feeling sad or “blue”, Black men may describe experiencing anger, frustration, and agitation [36]. This contextual, culturally-rich understanding may go undetected by, or be uncomfortable for health professionals [37], raising important questions about the ways health professionals might intervene.

Just as mental health is a complex facet interwoven throughout the lives of Black men, so are masculinities, or their definitions of manhood [22,24]. Because men’s definitions of manhood may vary by type, across different cultures, and evolve over time, I use the term “masculinities” to convey this variability. Culturally rich accounts of men’s definitions of manhood tend to be historically hegemonic, which are linked to poor mental health [20]. Yet, little is known about the extent to which Black men subscribe to conceptualizations of masculinities and mental health, collectively. We know from previous literature that a man’s conformity (or nonconformity) to certain race, culture, and gender norms may either permit or prohibit his adherence to a shared understanding of masculinities and mental health [7,20]. We also know that conformity to masculine norms has both advantages and disadvantages for young Black men. It promotes acceptance from some groups and provides social and economic rewards, yet it may also lead to emotional distancing and interpersonal dominance in relationships [7,20]. Studies suggest some marginalized men may also subscribe to a “dark side” of masculinity, in which they are active participants in violence, risky behaviors, and absent fathering, as a way to develop and redefine their own “traditional” masculine norms [38,39,40,41,42].

Social support among boys and men has direct implications for their social functioning. In more recent years, the use of internet-based social support groups has quadrupled [43,44,45,46], and the anonymity and confidentiality they offer increases the potential of self-disclosure and encourages honesty and intimacy among participants when discussing stigmatizing topics, such as those related to health [47,48]. Internet support groups are typically self-help in nature, and studies have reported positive outcomes from their use—most notably, that they decrease depression over the life span [49,50]. Internet-based communities and networks provide young Black men with a safe space [32,33,51] that is accessible 24 h a day, 7 days a week, for communicating about topics that would otherwise make face-to-face groups of young men uncomfortable (e.g., stress, racism, and sexism) [49,50,51,52,53,54]. Another appealing feature of an internet-based group format is that it is less spontaneous than face-to-face encounters, thereby allowing participants to think about their responses before sharing them with other group participants [48]. Men of all races who participate in internet-based social support groups tend to be more empathetic to the problems experienced by other men. This experience is not often shared, or welcomed, by all-male support groups that occur in face-to-face settings [48,49,50,51,52,53,54].

## 3. The YBMen Project

### 3.1. Description of the YBMen Project Intervention

The Young Black Men, Masculinities, and Mental Health (YBMen) project [32,33,55] was born in 2008 and launched in 2014 as a 6-week, Facebook-based mental health education and social support intervention for young Black men that uses gender-specific, age-appropriate, and culturally-sensitive prompts from popular culture (e.g., song lyrics, photos, YouTube videos, news headlines) to educate participants about the importance of mental health, progressive definitions of manhood, and social support [33]. Since its inception, the social media platforms Facebook and Instagram have been used to reach Black men and boys with relevant content. The research design for the YBMen project is a quasi-experimental, convergent mixed methods design in which the YBMen project team partners with school and commnity partners to adapt the program content to specific subgroups of Black men and boys. Our team members work with our partners to recruit participants using a variety of techniques including, but not limited to face-to-face encounters, flyers, social media sites, emails, and word of mouth.

The YBMen project is grounded in techniques focused on action planning and feedback, group problem-solving, and individual decision-making, to improve health outcomes [32,33]. These techniques are expanded during each week’s topic. During week 1, participants are introduced to the YBMen group and oriented to the style and format of the intervention (Week 1 goal: To get participants acclimated to the social media group, and build an online “community”). During week 2, participants receive content on Black masculinity (Week 2 goal: To familiarize participants with the idea that multiple masculinities exist for men and boys beyond rigidly defined gender roles). During week 3 we share mental health education and awareness materials (Week 3 goal: To imcrease participants’ mental health literacy). Week 4 covers overall health, wellbeing, and coping (Week 4 goal: To teach participants about topics related to their overall health, well-being, and coping strategies). Week 5 covers the importance of social support (Week 5 goal: To encourage participants to engage in more social support activities and build and sustain healthy social relationships). Week 6 includes a review of previous content and aims to establish individual and group sustainability plans moving forward (Week 6 goal: To review content from previous weeks and plan next steps for the group beyond the work done in the YBMen intervention).

### 3.2. The YBMen Project: Intervention Goals and Objectives

Health promotion interventions designed for Black men as they transition to and through early adulthood provide a unique opportunity to intervene at a time when they are susceptible to stress, depression, and risky health behaviors that contribute to early morbidity and mortality. While challenges persist when engaging Black men in traditional health interventions, the use of internet-based health education programs for Black men is promising. As a group, Black men tend to report high rates of social media use. The YBMen project’s social media intervention serves as a platform through which young Black men can enrich their mental health, transform their gender norms, and engage in healthy social relationships. The YBMen project intervention seeks to: (a) Provide useful mental health, masculinities, and social support resources to Black men, (b) share these resources through gender-specific, age-approprioate, and culturally-sensitive mechanisms, and (c) build capacity to change the narrative of mental health, manhood, and social support for Black men.

### 3.3. The YBMen Project Conceptual Framework

The YBMen project conceptual framework originated from grounded theory and systematic reviews that synthesized the influence of social determinants on the mental health of Black men over the life course [7,8,16,17]. This foundation serves as a lens through which the YBMen project is designed, implemented, and evaluated, as well as an umbrella for the synergistic integration of social cognitive theory [56] and theories of social networks and social support [57], which have helped the YBMen framework to evolve over time. The current conceptual framework focuses on understanding young Black men in the context of their lived experiences and how these experiences influence mental health, manhood, and social support. The grounded work on the social determinants of Black men’s mental health has been published elsewhere [7,8,16,17]. Thus, here, I describe social cognitive theory and theories of social networks and social support and how they are operationalized in the YBMen project.

First, social cognitive theory (SCT) (Table 1) explains how people acquire and maintain certain behavioral patterns, while also providing the basis for intervention strategies [56]. Deisnging, implementing, and evaluating behavioral change depends on the environment, people, and behavior because each one is constantly influencing the others. Behavior is not simply the result of the environment and the person, just as the environment is not simply the result of the person and behavior [57]. A basic premise of SCT is that people not only learn through their own experiences but also by observing the actions of others and the results of those actions. Personal factors include a person’s capacity to symbolize behavior, to anticipate the outcomes of behavior, to learn by observing others, to have confidence in performing a behavior (including overcoming any barriers to performing the behavior), to self-regulate behavior, and to reflect and analyze experiences [58].

SCT is applicable to the YBMen project because the theory involves cognitive and emotional facets of behavior for understanding behavioral change. For the purposes of the YBMen project intervention, six components of the SCT are applied: Behavioral capacity, expectations, observational learning, reinforcement, reciprocal determinism, and self-efficacy. *Behavioral capacity* is defined as the knowledge and skills to influence behaviors, and this is operationalized through the YBMen project by educating its participants on individual and collective definitions of mental health, manhood, and social support and empowering their roles as change agents. *Expectations* are the anticipatory outcomes of a behavior. YBMen participants can share their experiences and expectations with behavioral outcomes as they fully engage with content that addresses mental health, manhood, and social support throughout the program.

*Observational learning* is a behavioral acquisition that occurs by watching the actions and outcomes of others’ behaviors. In the YBMen project, participants discuss their experiences and the outcomes of those experiences with other participants. They also incorporate role models into these discussions to illustrate their targeted behavior. *Reinforcement* is a response to a person’s behavior that either decreases or increases its reoccurrence. When delivering the YBMen intervention, positive, group-affirming behaviors that challenge hegemony and encourage participants to think more critically about positive mental health, manhood, and social support are reinforced. *Reciprocal determinism* is the dynamic interaction between a person, their behavior, and the environment in which the behavior occurs. In the YBMen project, participants work together to disentangle the hegemonic and mainstream narratives associated with mental health, manhood, and social support, and work to change how they engage with these narratives. Finally, *self-efficacy* is a person’s confidence in performing a behavior. With the YBMen project, tools of persuasion, encouragement, and strengths-based affirmation are used to support participants’ confidence and thinking trajectories.

In addition to SCT, the YBMen project is guided by theories of social networks and social support (SNSS) [57], which are not theories per se but *“…concepts that describe the structure, processes, and functions of social relationships”* (pg. 193). Theories of SNSS (Table 2) are rooted in the advantages of examining the effect of social relationships on health. This is depicted in three very important ways. First, social network approaches can incorporate characteristics of social relationships beyond that of social support. Second, including both theories of social networks and social support allows for an opportunity to focus on one relationship at a time (i.e., social support) as well as how changes in one relationship affect other relationships (i.e., social network). Finally, a SNSS approach enables the exploration of how structural network characteristics influence the quantity and quality of the social support exchanged between the people involved.

**Figure 1:**
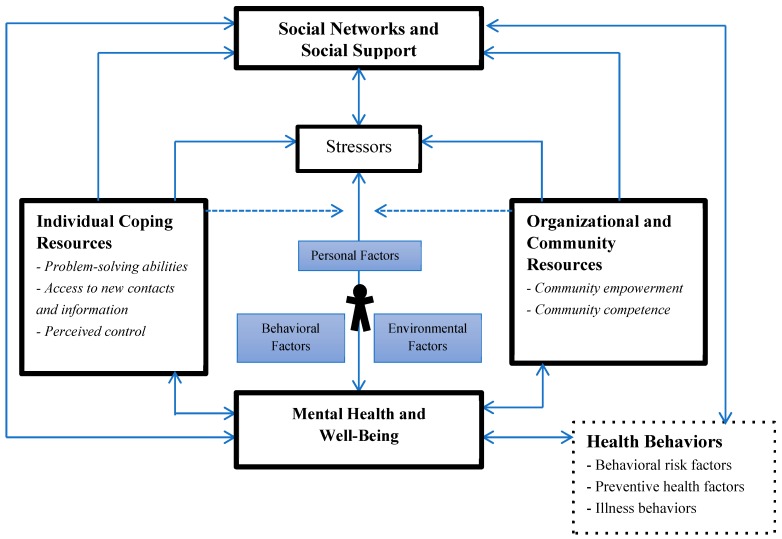
The integration of social cognitive theory (SCT) and theories of social networks and social support (SNSS) that inform the YBMen project conceptual framework.

With theories of SNSS, resources at both the individual and community levels may have direct health-enhancing effects and may also diminish the negative effects on health due to exposure to stressors. When people experience those stressors, having enhanced individual or community resources increases the likelihood that stressors will be handled or coped with in a way that reduces adverse health consequences both short-term and long-term. This effect is called the “buffering effect” and is illustrated by the dotted lines in Figure 1. The pathway from social networks and social support to health behaviors makes it explicit that social networks and social support may affect the incidence of and recovery from disease.

In the application of SCT and theories of SNSS to the YBMen project, our team: (1) Carefully describes intervention activities to partners and participants; (2) monitors the effects of the intervention activities on the quality of social support both delivered and received; and (3) assesses changes in knowledge, health behaviors, community capacity, and health status. The interplay between the personal, behavioral, and environmental factors of the SCT are interpreted in light of the social networks and social support experiences of young Black men (Figure 1). These factors occur in the context of the social networks and social support in which they engage as a function of their mental health, well-being, and stress. The YBMen project has tested the efficacy and effectiveness of an intervention that will enable young Black men to participate in a social media-based intervention that circumvents social and cultural barriers that impede their development during face-to-face intervention settings [32,33]. For example, our programs have been implemented among well-established groups of Black men and boys, which would be considered ideal, controlled settings (i.e., efficacy) as well as among more informal groups of Black men, which would be considered “real world” settions (i.e., effectiveness).

## 4. Reflections: Looking Back at What We Have Done

### 4.1. The Importance of Including Multiple Measures to Understand Black Men and Mental Health

Previous work has uncovered inconsistencies in how Black men report symptoms on current mental health measures and how Black men describe and express their own mental health. For instance, previous studies comparing the depressive symptomatology of Black college men at a predominately white institution (PWI) and a historically Black college/university (HBCU) revealed similar rates of depression but differences in the types of stressors they experienced by institution. While PWI men reported racism, alienation, and isolation on campus, men at the HBCU reported dysfunctional families, money, and stress off campus [16,23]. Research has also found unique associations between social determinants and depressive symptoms, serious psychological distress, and major depressive disorders for Black men across different age groups [4,8]. Similarly, Shepard Payne found differences in symptom expression for African American men during their interactions with clinicians [36]. Other studies identified population characteristics that included measures of masculinity, men of color, and a measure of mental or physical health behavior [18]. Mental health was the most common outcome in the studies identified, followed by health behavior. Findings supported the need for future research on: (1) The centrality of masculinity versus racial and gender identity in men’s identities and health; (2) frameworks that underscore the relationship between masculinity and health outcomes; and (3) conceptions of masculinity that are most relevant to and associated with health behaviors and outcomes (e.g., stoicism is associated with poor health) [18]. In addition to these recommendations, I would emphasize the need for qualitative, quantitative, and mixed methods to further understand the experiences of and mechanisms that shape Black men’s mental health.

### 4.2. The Importance of Social Media and Online Social Support in Programs for Black Men

Previous scholars have examined the relationship between perceived social capital and specific social media-enabled communication behaviors using survey data from samples of U.S. adults [46,59,60,61]. For example, drawing upon scholarship on social capital and relationship maintenance, Ellison and others [59,60,61,62] examined the role of Facebook attention-signaling behaviors in shaping perceived access to resources in one’s network as measured by bridging social capital. Comparable studies have demonstrated the success of using online social support, generally [31], and specifically for Black men with psychological distress [32,33]. Participation in online support groups can serve as preventative methods for poor mental health over the life span [49,50,51,52]. The use of online social support groups has quadrupled in the past decade; therefore, an online community provides Black men with a safe space [32] that is accessible, convenient, and free. Notwithstanding, it is also important to consider the global reach of social media and online social support, especially because Black men across the globe are experiencing mental health challenges in their own economic and social contexts [63].

### 4.3. The Importance of Prioritizing Behavioral Health Interventions for Young Black Men

Research has been instrumental in the development and refinement of conceptual frameworks for how to engage in mental health research and practice efforts with Black youth; moreover, fewer have focused solely on Black men (see References [7,8,18,64] for exceptions). Tate studied sociocultural factors that influence risky behaviors among African American adolescents and uncovered the importance of developing interventions that explore gender, socioeconomic status, and residential status and how these variables are associated with health in African American young adults in urban and suburban settings [65,66]. Expanding behavioral health interventions with Black people to include a gender focus will enhance the future trajectory of this work. Building on the foundation of previous studies in this area that underscores the importance of race, gender, class, masculine norms, and social context in research and practice with Black men, future work should prioritize studies that develop and test behavioral interventions for Black men who experience psychological distress [7,31,32,33]. Additionally, it is important to consider the role of ethnic/genetic predispositions to mental disorders and how they intersect with social constructionist theories and stereotypes in behavioral health interventions that are created for Black men. Collectively, these scholarly contributions will advance this area by offering practical strategies [64] to improve Black men’s mental health and wellbeing [16,17,18,32,33,34].

### 4.4. The Importance of Evaluation for Interventions Aimed at Boys and Men of Color

The evaluation framework for the YBMen project includes process, impact, and outcome evaluations. The evaluations are based on the assertion that effective interventions for boys and young men of color should be informed by theories and models that identify the strengths and positive attributes of men as well as the risks [32]. In the case of the YBMen project, an integration of the SCT and theories of SNSS have allowed investigators to holistically examine effects of the intervention at individual, interpersonal, and system levels. Program evaluations conducted from multilevel viewpoints provide insight into the various intersecting variables that impact individual and community change. Likewise, the multidimensional efforts aimed at designing, implementing, and evaluating the YBMen project were born out of research on the need for gender-specific healthcare [64]; the empowerment of men [34]; and the importance of including culturally-sensitive materials in the development of strategies designed to encourage self-efficacy and personal mastery of health for boys and young men of color. The cyclical process for learning and using what we learn to achieve the proposed goals and objectives occurs at multiple levels.

## 5. Projections: Looking Ahead to the Future

The racially motivated tensions across the United States over several decades have underscored the life stressors faced by young Black men and boys, including racism, discrimination, unemployment, and poverty. These life stressors can put these subgroups at risk for poor mental health over time. When developing mental health promotion programs for Black men and boys, psychoeducational interventions have considerable advantages over other types of interventions. Young Black men often hold negative opinions about clinical interventions which, when coupled with the fact that they often lack medical insurance to offset the cost of medical evaluations [67,68], stand as formidable obstacles to the provision of care in this group.

The World Health Organization defines social determinants as the conditions in which people are born, grow, live, work, and age [69]. This postive interpretation of social determinants is promising but leads to challenges for marginalized groups because these conditions are shaped by the distribution of money, power, and resources at global, national, and local levels; many marginalized groups lack the material means or social capital to benefit from their conditions. For instance, some scholars argue that social determinants are primarily responsible for the disparities that exist with regard to the mental health of Black men. However, initiatives like the YBMen project underscore social determinants of mental health for Black men and boys by considering their lived experiences, seeking to understand their definitions of manhood, and offering a platform to enhance the likelihood that this group will seek social support and help when in need [32,33]. Namely, the social determinants that are addressed via the YBMen project are varied and include employment and socioeconomic status, kinship and social support, masculinities, stress (in the context of racism and discrimination, violence, and gendered stress), and incarceration [7]—all in an effort to improve the living, learning, and thriving of young Black men.

The YBMen project has been successfully implemented across multiple sites in the Midwest USA; findings demonstrate the potential for adaptation and expansion. Building on the success of previous research, the goal of future iterations of the YBMen project will be to (1) culturally target a mental health campaign for Black men and boys, (2) adapt and implement the YBMen intervention for other subgroups of Black men and boys, and (3) manualize the program for expansion and dissemination. In more recent iterations of the project, we lowered the eligibility age to 13 to provide more early intervention opportunities for Black males. This way, building a mental health campaign and improving Black men’s mental health, conformity to traditional masculine norms, and social support can begin earlier in their lives. Teams of researchers, practitioners, and community stakeholders are well-positioned to develop and test culturally-sensitive, age-appropriate, and gender-specific resources for Black men and use these innovative strategies to build capacity and ensure sustainability that will result in fewer mental health challenges among Black males as they live, learn, and thrive.

The social and economic hardship Black men face—and marginalized roles within their families and communities—restricts their ability to establish and maintain positive mental health and wellbeing as they age [4,5,6,7,8,9,10,11,12,13,14]. From these challenges and the history of racial discrimination Black men experience early in their lives, some scholars have noted that masculine norms for Black men are accentuated by masculine attributes such as strength, control, independence, and self-sufficiency [11,21]. This in turn creates a world where Black men are significantly less likely than girls and young women of color to seek help for their mental health problems. The YBMen project prioritizes mental health, treating masculinity as functional and adaptive, which allows Black men to be productive under a number of difficult conditions. For example, when it comes to seeking help for mental health challenges, Black men tend to avoid discussing their problems with others.

The YBMen project is a promising step in the right direction. Currently, it aims to to educate Black men and boys about the importance of mental health, progressive definitions of manhood, and social support [33]. It also provides participants with a language for how to talk about their challenges, as well as the path to seeking formal and informal help, should participants need it. The YBMen project underscores unique opportunities to use technology as a way to deliver mental health education and provide social support to Black men in a private, nonthreatening setting that promotes their mental health and well-being. However, is also underscores the importance of co-creating programs with interdisciplinary teams, including the subgroups of boys and men we want to serve. Including their feedback and recommendations into the programs we develop for them is necessary to truly have make an impact on their lives. For instance, participants in some of our previous iterations of the project mentioned the value of the online connection but that they would also appreciate some face-to-face time. Thus, future versions of the YBMen project will incorporate opportunitites for participants to meet face-to-face and discuss their online group activities. The potential impact of these connections could be inspiring for Black men and boys, particularly those who want to initiatie new and/or existing emancipatory and antiracism campaigns to evoke social change in their communities and acorss the globe.

## 6. Conclusions

A culturally adapted psychoeducational intervention for Black men delivered through a medium that is already integrated into their lifestyle, such as social media, is likely to be more acceptable and sustainable compared to other styles of health education. Further, researchers have recommended an intersectional approach to Black men’s mental health [7,18], that considers the intersection of multiple dimensions of socially-constructed identity, including race, gender, class, masculine norms, and context. Research also underscores the independent effects of each of these dimensions, but the current paper highlights the need to understand the unique ways in which these dimensions unite and influence the experience of mental health for young Black men. In addition, because the cultural environment is heterogeneous for Black men, there is a critical need for interventions that examine how social factors, cultural and behavioral factors, psychosocial exposures, and life course variables influence mental health and evolve over time.

## Figures and Tables

**Table 1:** Social cognitive theory (SCT) and how it is operationalized in the Young, Black Men, Masculinities, and Mental Health (YBMen) project.

SCT Concept	Definition	Application to the YBMen Project
Behavioral capacity	Knowledge and skills to influence behavior	Educate Ps on individual and collective definitions of mental health, manhood, and social support and empowering their role as change agents.
Expectations	Anticipatory outcomes of a behavior; Model positive outcomes of healthful behavior	Encourage Ps to describe positive and negative outcomes of their previous behaviors.
Observational learning	Behavioral acquisition that occurs by watching the actions and outcomes of others’ behavior; Include credible role models of the targeted behavior	Share Ps individual and collective experiences; Identify credible role models to emulate that help portray the experiences of Black men in society.
Reinforcement	Responses to a person’s behavior that increase or decrease the likelihood of reoccurrence; Promote self-initiated rewards and incentives	Offer praise to and between Ps; Encourage self-reward; Decrease possibility of negative responses that deter positive changes.
Reciprocal determinism	The dynamic interaction of the person, the behavior, and the environment in which the behavior is performed	Involve Ps and others in program adaptation; Work with Ps to consider multiple avenues to behavioral change including environmental, skill-based, and personal change.
Self-efficacy	The person’s confidence in performing a particular behavior; Approach behavioral change in small steps to ensure success	Work with Ps to acknowledge strengths; Use persuasion and encouragement; Behavior change occurs via small, achieveable milestones that Ps can master.

Ps = participants.

**Table 2:** Theories of social networks and social support (SNSS) and how they are operationalized in the YBMen project.

Concepts	Definitions	Application to the YBMen Project
Structural characteristics of social networks		
Reciprocity	Extent to which resources and support are both given and received in a relationship	Ps receive information from the program, but are also asked to share information with members of their group.
Intensity of strength	Extent to which social relationships offer emotional closeness	Some Ps know one another prior to the start of the group while others strengthen relationships while in the group.
Complexity	Extent to which social relationships serve many functions	Given the nature of the topics covered in the groups, the needs and expectations of Ps may expand
Formality	Extent to which social relationships exist in the context of organizational or institutional roles	Many Ps are students and/or members of social groups. Many of their group exchanges are in this context.
Density	Extent to which network members know and interact with each other	Some Ps see and interact with one another outside of YBMen; others restrict their contact to what occurs within the group.
Homogeneity	Extent to which network members are demographically similar	Many Ps are connected via race and gender and associated experiences. Other identifying characteristics emerge in the YBMen group.
Geographic dispersion	Extent to which network members live in close proximity to focal person	Due to the focus on community, facilitated by on online community, the idea of proximity is less emphasized.
Directionality	Extent to which members of the dyad share equal power and influence	Ps are on an ‘even’ playing field. This may influence their interaction with the YBMen group moderator, who remains anonymous throughout the program.
Functions of social networks		
Social Capital	Resources characterized by norms of reciprocity and social trust	Built by Ps in YBMen groups; particularly helpful when Ps vary by age, economic position, focus of study or work, identity, geographic area, etc.
Social Influence	Process by which thoughts and actions are changed by the actions of others	YBMen is based on social influence that helps Ps make informed decisions about how their mental health, manhood, and support are integrated.
Social undermining	Process by which others express negative affect or criticism or hinder one’s attainment of goals	Ps may be exposed to this based on group dynamics and individual contributions of Ps. Rapport-building early influences this concept.
Companionship	Sharing leisure or other activities with network	Regarded as a possible outcome of the YBMen program by Ps.
Social Support	Aid and assistance exchanged through social relationships and interpersonal transactions	A core aim of YBMen. Ps often come to the program interested in social support, and leave with a renewed appreciation for quality relationships.
Types of social support		
Emotional support	Expressions of empathy, love, trust, and caring	Evident in group once Ps build rapport and normalize men’s emotions.
Instrumental support	Tangible aid and services	Not the focus of the group, but may occur once networks are built and connections are made.
Informational support	Advice, suggestions, and information	Evident in group once Ps build rapport and realize they have contributions to make.
Appraisal support	Information that is useful for self-evaluation	Ongoing introspection among Ps in the YBMen groups.

Ps = participants.
